# Integrative analysis revealed a correlation of PIAS family genes expression with prognosis, immunomodulation and chemotherapy

**DOI:** 10.1186/s40001-024-01795-7

**Published:** 2024-03-25

**Authors:** Qiqi Zhang, Junkui Zhang, Tianyi Lan, Jiayue He, Bin Lei, Hongnan Wang, Zhiqiang Mei, Chaoxiang Lv

**Affiliations:** 1 The Research Center for Preclinical Medicine, Southwest Medical University, Luzhou, 646000 Sichuan China; 2 Pharmaceutical Institute, Henan University, Kaifeng, 475004 China; 3 College of Integrative Chinese and Western Medicine, Southwest Medical University, Luzhou, 646000 Sichuan China

**Keywords:** PIAS, Tumor microenvironment, Immune cell infiltration, Single-cell RNA-Seq, Immunotherapy

## Abstract

**Background:**

Protein inhibitor of activated STATs (PIAS) has pleiotropic biological effects, such as protein post-translational modification, transcriptional coregulation and gene editing. It is reported that PIAS family genes are also correlated with immune cells infiltration in cancers that highlights their unnoticed biological role in tumor progression. However, the relationship of their expression with prognosis, immune cell infiltration, tumor microenvironment, and immunotherapy in pan-cancer has been rarely reported.

**Methods:**

The multi-omics data were used to investigate the expression level of PIAS family members in pan-cancer, and the prognostic value of their expression in different tumors was analyzed by univariate Cox regression and Kaplan–Meier. Correlation analysis was used to investigate the relationship of PIAS gene expression with tumor microenvironment, immune infiltrating subtypes, stemness score and drug sensitivity. In addition, we also used wound healing and transwell assays to verify the biological effects of PIAS family gene expression on invasion and metastasis of HCC cells.

**Results:**

We found that PIAS family genes expression is significantly heterogeneous in tumors by multi-genomic analysis, and associated with poor prognosis in patients with multiple types of cancer. Furthermore, we also found that genetic alterations of PIAS family genes were not only common in different types of human tumors, but were also significantly associated with disease-free survival (DFS) across pan-cancer. Single-cell analysis revealed that PIAS family genes were mainly distributed in monocytes/macrophages. Additionally, we also found that their expression was associated with tumor microenvironment (including stromal cells and immune cells) and stemness score (DNAss and RNAss). Drug sensitivity analysis showed that PIAS family genes were able to predict the response to chemotherapy and immunotherapy. PIAS family genes expression is closely related to tumor metastasis, especially *PIAS3*. High PIAS3 expression significantly promotes the migration and invasion of liver cancer cell lines (HCC-LM3 and MHCC97-H).

**Conclusions:**

Taking together, these findings contribute to determine whether the PIAS family genes are a potential oncogenic target gene, which have important contribution for the development of cancer immunotherapy.

## Introduction

Cancer is the most common cause of death in the worldwide, and poses a significant obstacle to global public health security and quality of life. According to the American Cancer Society, the number of new cancer cases and deaths is expected to rise to 19.3 million and 10.0 million, respectively, by 2020 [1]. Despite the diagnosis and treatment of cancers has been made great progress with rapid advances in medical technology, the prognosis for patients is still discouraging. As alternatives to classical anti-cancer therapy, targeted treatment and immune checkpoint blocking therapy have been shown to be effective for some types of human cancer [2]. However, only a minority of tumors have targetable molecules, and the efficacy of immunotherapy is far from satisfactory [3]. Thus, exploring the relationship of gene expression in tumors with prognosis, immune cell infiltration and chemotherapy sensitivity is beneficial to evaluate the biological role of targeted molecules, as well as gain insight into the underlying mechanisms of tumorgenesis.

As protein inhibitor of activated STATs (signal transducer and activator of transcription), PIAS family genes have pleiotropic biological effects, including protein post-translational modification, transcriptional coregulation and gene editing [4]. Recently, increasing evidences have shown that PIAS family genes play important roles in physiological and pathological processes, and their expression is closely related to human diseases, especially cancer [5–7]. For example, downregulation of PIAS1 inhibits the differentiation of tumor cells in liver cancer [8], and it also serves as a biomarker to distinguish colon cancer from adenomas [9]. In addition, PIAS1 is also a potential biomarker indicating stress susceptibility [10]. In a variety of tumors, the expression of PIAS3 protein is upregulated, including lung, colorectal, breast, and prostate cancers [11–14]. PIAS4 is closely related to the occurrence and progression of certain types of human cancers (such as pancreatic cancer and liver cancer) [15, 16]. These findings indicate that PIAS family genes are broadly involved multiple biological functions among human cancers.

However, it is rarely reported that the effect of PIAS family genes with on the immune system and their relationship with prognosis, tumor microenvironment (TME), and immunotherapy in pan-cancer. Here, we revealed the expression patterns of PIAS family genes in human pan-caner and explored the important contribution of their genetic alterations in influencing patient outcomes. Besides, we also analyzed the correlation of PIAS family genes with immune cell infiltration, immune subtype and tumor metastasis, particularly in kidney renal papillary cell carcinoma (KIRP) and liver hepatocellular carcinoma (LIHC). These findings are conducive to understanding the biological roles and regulatory mechanism of PIAS family genes in tumor progression, which have important contribution for the further investigation of cancer immunotherapy.

## Materials and methods

### Data acquisition and PIAS family genes expression analysis

We mined 11,069 patient data from the cancer genome atlas (TCGA) database for thirty-three types of cancer using UCSC Xena online platform (https://xenabrowser.net/datapages/), including RNA expression (HTSeq-FPKM), clinical parameters, immune subtypes, and stemness scores datasets [17]. For pan-tumor analysis, R-package "pheatmap" was used to compare the expression of PIAS family genes in cancerous tissues with para-cancerous or normal tissues, and R-package ‘‘corrplot’’ and ‘‘ggplot2’’ were respectively implemented for correlation analysis and visual analysis of the results. TNM plotter (https://tnmplot.com/analysis/) was used to investigate differences in PIAS family genes expression between tumor, metastatic, and normal tissues [18]. The wilcoxon rank-sum test was used for inter-group statistical analysis, and p-value less than 0.05 was considered to be statistically significant. ‘‘*’’ means p < 0.05, ‘‘**’’ means p < 0.01, and ‘‘***’’ means p < 0.001.

### Cox regression and survival analysis across pan-cancer

The Cox univariate regression analysis was performed to investigate the role of PIAS gene expression level in the risk of prognosis. The samples were divided into high- and low-expression groups according to the median level of PIAS family genes expression. After that, the forest plots were plotted using ‘‘forestplot’’ package in R software (version 4.3.0). For survival analysis, we used different databases to determine the relationship between PIAS family genes expression and clinical outcomes, including GEPIA2 (an online database, http://gepia.cancer-pku.cn/index.html), Kaplan–Meier plotter (a public online platform, http://kmplot.com/analysis/) and PrognoScan (http://dna00.bio.kyutech.ac.jp/PrognoScan/index.html). GEPIA2 was used to analyze the correlation between PIAS family genes expression and overall survival (OS) in 33 different cancer types. Kaplan–Meier plotter database was performed to evaluate the prognostic significance of PIAS family genes expression in pan-cancer. PrognoScan database was used to identify the correlation of PIAS family genes expression with clinical outcome, including OS (overall survival), DFS (disease free survival), DSS (disease specific survival), and RFS (relapse free survival).

### Genetic alteration analysis of PIAS family genes in pan-cancer

Considering the integration of genetic alteration data, we use the public database cBioPortal (https://www.cbioportal.org/) to explore PIAS family genes alterations in TCGA pan-cancer samples [19]. The ‘‘Cancer Type Summary’’ module was selected to analyze their alteration landscape across pan-cancer. The ‘‘Mutation’’ module was performed to a mutation site plots for PIAS family genes. To explore the relationship between genetic alterations in the PIAS family genes and clinical outcomes, we divided patients into unaltered and altered groups. The survival curve was generated by the ‘‘Compare/Survive’’ module.

### Analysis of TME, immune subtypes and stemness score in pan-cancer

We evaluated the correlation of PIAS family genes expression with TME using the ESTIMATE algorithm that was presented in the form of stromal score and immune score. Subsequently, pearson correlation coefficient was used to examine the association between PIAS family genes expression and TME. For the immune subtype analysis, we first downloaded six immune subtypes (C1 ~ C6) from the UCSC Xena database. And then, box plots were used to analyze the expression of PIAS family genes by using R software in different immune subtypes. R-packages ‘‘cor. Test’’ was used to detect the correlation of PIAS family genes expression with DNAss and RNAss. R-packages ‘‘ggplot2’’ was implemented for visual analysis of the results, and p-value less than 0.05 was considered to be statistically significant.

### Immune correlation and single-cell sequencing analysis

We applied the public database TIMER (https://cistrome.shinyapps.io/timer/) to evaluate the correlation of PIAS family genes expression with immune cells. The abundances of six immune cell infiltrates are estimated by TIMER algorithm, including B cell, CD4^+^ T cell, CD8^+^ T cell, neutrophils, macrophages, and dendritic cells (DC) [20]. The correlation between PIAS family genes expression and immune cells was examined by pearson correlation coefficient. R-packages ‘‘pheatmap’’, ‘‘ggpubr’’ and ‘‘limma’’ were applied for analysis, and the results were displayed in the heatmap. For single-cell sequencing analysis, we selected tumor immune single-cell Hub 2 (TISCH2) database (http://tisch.comp-genomics.org/) to investigate the association of gene expression with immune cells [21]. PIAS family genes expression at the single-cell level in the KIRP_GSE159913 and LIHC_GSE16635 datasets was visualized with the ‘‘dataset’’ module. The wilcoxon rank-sum test was used for inter-group statistical analysis, and p-value less than 0.05 was considered to be statistically significant. ‘‘*’’ means p < 0.05, ‘‘**’’ means p < 0.01, and ‘‘***’’ means p < 0.001.

### Drug sensitivity analysis

From CellMiner^™^ database (http://discover.nci.nih.gov/cellminer/home.do), we collected the sensitivity processing data of different drugs and RNAseq expression data of PIAS family gene [22]. The RNAseq expression data were divided into high- and low-expression groups according to the median level of PIAS family genes expression. Subsequently, R-package ‘‘ggplot2’’ was used for analysis, and the results were presented in box plots. The wilcoxon rank-sum test was used for inter-group statistical analysis, and p-value less than 0.05 was considered to be statistically significant. ‘‘*’’ means p < 0.05, ‘‘**’’ means p < 0.01, and ‘‘***’’ means p < 0.001.

### Cell culture and cell transfection

Human hepatocellular carcinoma cell lines HCC-LM3 and MHCC97-H were derived from the Medical Basic Research Center of Southwest Medical University. All cells were cultured at 37 ℃ and 5% CO_2_ in dulbecco's modified eagle medium (DMEM, Gibco, C11995500BT) supplemented with 10% bovine serum (Gibco, 10091148) and 1% penicillin–streptomycin (Gibco, 15140122). For plasmid transfection, full-length PIAS3 was subcloned into pcDNA3.1( +) vector by BamH1 and XbaI. Subsequently, transient transfection was performed according to lipofectamine 3000 transfection reagent (Invitrogen, L3000015) protocol after the cells were inoculated on 6-well or 12-well plates.

### Quantitative real-time PCR and western blotting analysis

Quantitative analysis of *PIAS3* and *GAPDH* (loading control) mRNA levels was performed by qRT-PCR method using a 2 × Power SYBR Premix Ex TaqTM (TaKaRa Bio INC, Japan) in a Bio-Rad iCycler & iQ qRT-PCR systems (Bio-Rad, Hercules, CA, USA). qRT-PCR for PIAS3 mRNA (forward: 5ʹ—TTTGTCAAGGTCAATGGGAAAC—3ʹ and reverse: 5ʹ—CGAACTCAGATGACCAATTGAC—3ʹ) was performed. GAPDH mRNAA (forward: 5ʹ—GTCTCCTCTGACTTCAACAGCG —3ʹ and reverse: 5ʹ—ACCACCCTGTTGCTGTAGCCAA—3ʹ) was used as the internal reference. For Western blotting, the cells were collected and the protein were blotted onto polyvinylidene fluoride (PVDF) after using 10% SDS-PAGE separation. After that, the membrane was blocked with 5% bovine serum albumin (BSA, Sigma, CAS, NO: 9048-46-8) in Tris-buffered saline with Tween 20 (TBST) before incubation with specific antibodies at 4 ℃ overnight. Then, the membranes were incubated with the secondary antibody for 2 h at room temperature (15 ~ 30 °C) before the blot samples was imprinted using an Easysee Western Blot Kit (Transgene, Alsace, France).

### Immunohistochemical staining

From December 2023 to February 2024, a total of 6 liver hepatocellular carcinoma (LIHC) specimens and corresponding para-carcinoma tissue or normal specimens were obtained from the Affiliated Hospital of Traditional Chinese Medicine of Southwest Medical University. Immunohistochemical analysis was conducted using the special antibody (PA5-116,023, Invitrogen, USA). All specimens were clinically and histologically diagnosed as LIHC. These specimens were stained in a blinded manner by pathologists, and the representative pictures are displayed. Additionally, we also received the immunohistochemical images of PIAS3 from the Human Protein Atlas (https://www.proteinatlas.org/) online-database.

### Wound healing, cell migration and cell invasion assay

Transfected cells incubated in 6-well plates were scraped with a 200 μl pipette tip in a straight line, and rinsed with PBS 3 times. After that, the cell migration data were observed under an inverted microscope at 0, 12 and 24 h after incubation. In the migration assay, the cells were washed three times with phosphate buffer saline. The cells were subsequently mixed with serum-free medium in the upper cavity, and 700 μL of cell medium containing 10% to 20% serum was added to the lower cavity. For the invasion experiment, the steps were the same as for the migration assay, except that the upper chamber was coated with matrigel diluted with serum-free medium 1:8 before inoculation. The cells were fixed after incubation for 48 h, and stained before being photographed.

### Statistical analysis of data

Statistical analysis of the experiments was performed using GraphPad_Prism (version 8.0.2.263). The whole data are presented as the mean ± standard deviation (SD) from at least three separate experiments. T-test was performed to compare differences between two groups. p < 0.05 was considered statistically significant, ‘‘*’’ means p < 0.05, ‘‘**’’ means p < 0.01, and ‘‘***’’ means p < 0.001.

## Results

### Expression of PIAS family genes in pan-cancer data

**Fig. 1:**
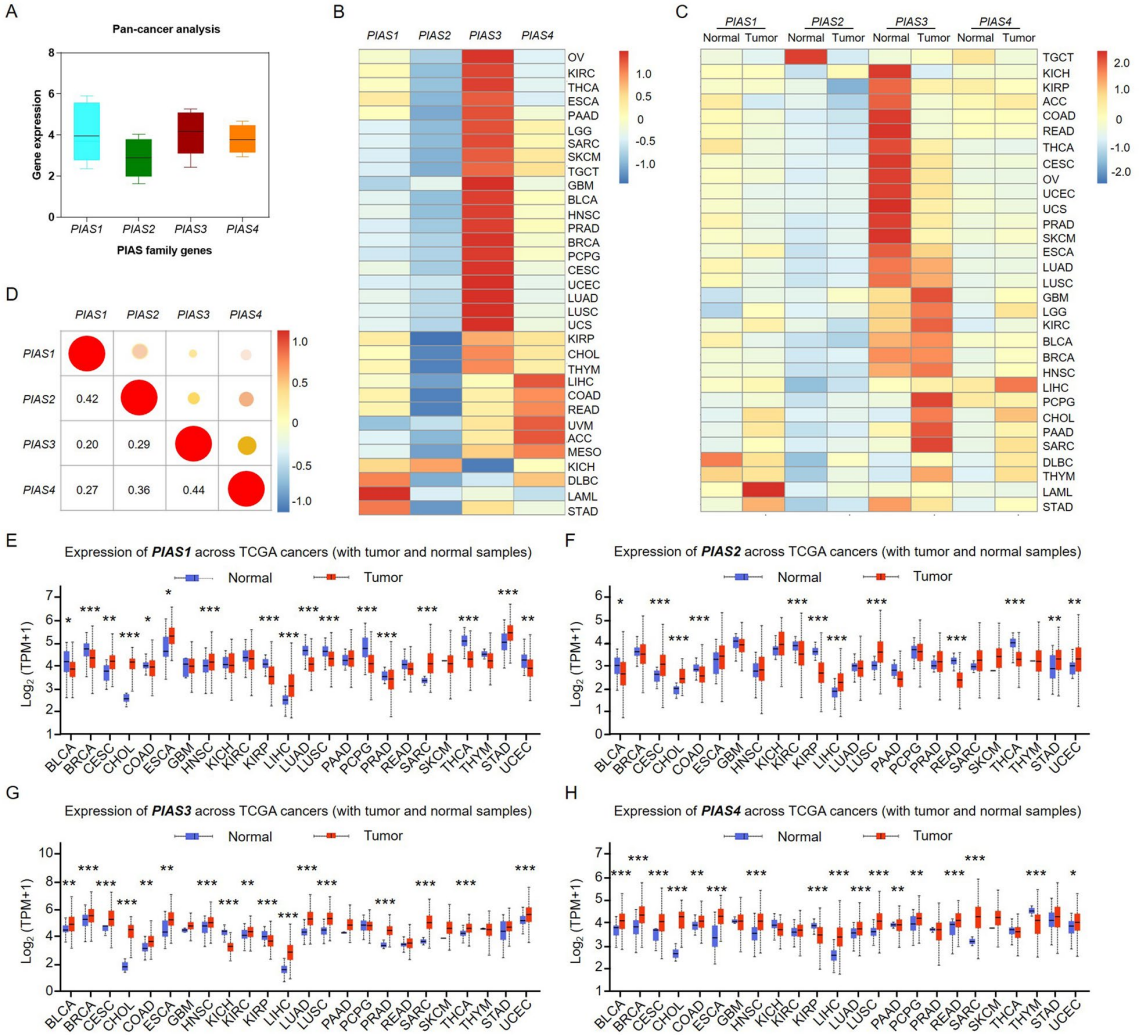
Expression patterns and correlation analysis of PIAS family genes in pan-cancer. **A** PIAS family genes expression in thirty-three different tumor types. **B** The expression level of PIAS family genes in different types of TCGA tumors. **C** Heat maps showed differences in PIAS family gene expression between tumor tissue and adjacent or normal tissue. **D** Correlation of PIAS family genes expression in pan-cancer. The expression level of PIAS family genes in different cancer types, as well as para-carcinoma or normal tissue. **E** *PIAS1*, **F** *PIAS2*, **G** *PIAS3*, **H** *PIAS4*. The red box represents tumor tissue, and the blue box represents para-carcinoma or normal tissue. *p < 0.05, **p < 0.01, ***p < 0.001

To explore the expression level of PIAS family genes in human cancers, we evaluated their expression patterns in thirty-three types of human cancers by using the TCGA databases (Fig. 1A). The results showed that *PIAS3* was the highest in tumors, while *PIAS2* had the lowest expression (Fig. 1B). By examining the expression level of PIAS family genes in tumor and para-cancerous or normal tissues, we found that their expression was heterogeneous (Fig. 1C). Correlation analysis showed that *PIAS1* had the highest positive correlation with *PIAS2*, while *PIAS3* had the highest positive correlation with *PIAS4* (Fig. 1D). Subsequently, we further analyzed the expression of PIAS family genes in different TCGA tumors (Table 1). Compared with normal tissues, *PIAS1* expression was significantly elevated in CESC, CHOL, ESCA, HNSC, LIHC, SARC, and STAD (Fig. 1E). Expression of *PIAS2* was significantly upregulated in CESC, CHOL, LIHC, LUSC, STAD, and UCEC (Fig. 1F). During the analysis of PIAS family gene expression, we also found that *PIAS3* was significantly higher expression in BLCA, BRCA, CESC, CHOL, COAD, ESCA, HNSC, KIRC, LIHC, LUAD, LUSC, PRAD, SARC, THCA, and UCEC (Fig. 1G). Besides, *PIAS4* was significantly higher expression in BLCA, BRCA, CESC, CHOL, COAD, ESCA, HNSC, LIHC, LUAD, LUSC, PAAD, PCPG, READ, SARC, and UCEC (Fig. 1H). These findings indicated that PIAS family members expression in different tumors was heterogeneous, which also suggested that they had some relevant biological functions and regulatory mechanisms for tumor progression.

**Table 1:**
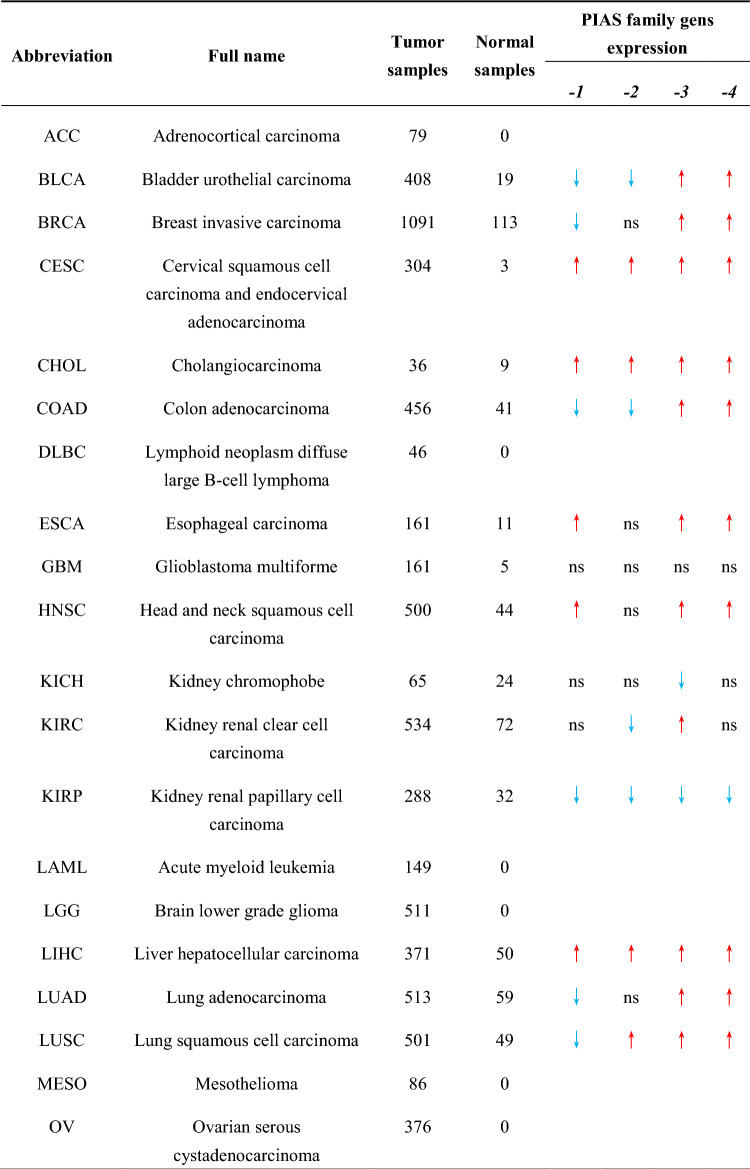

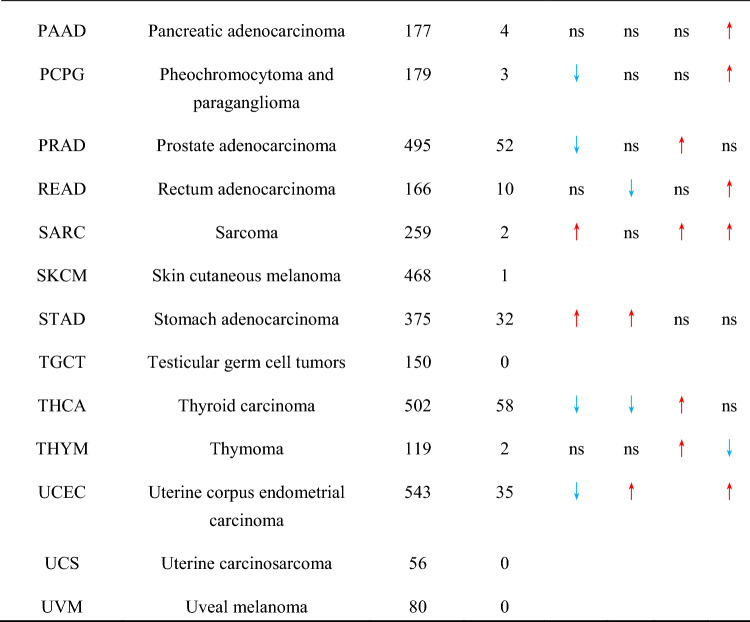
Pan-cancer data acquired from TCGA database

### Prognostic value of PIAS family genes across pan-cancers

**Fig. 2:**
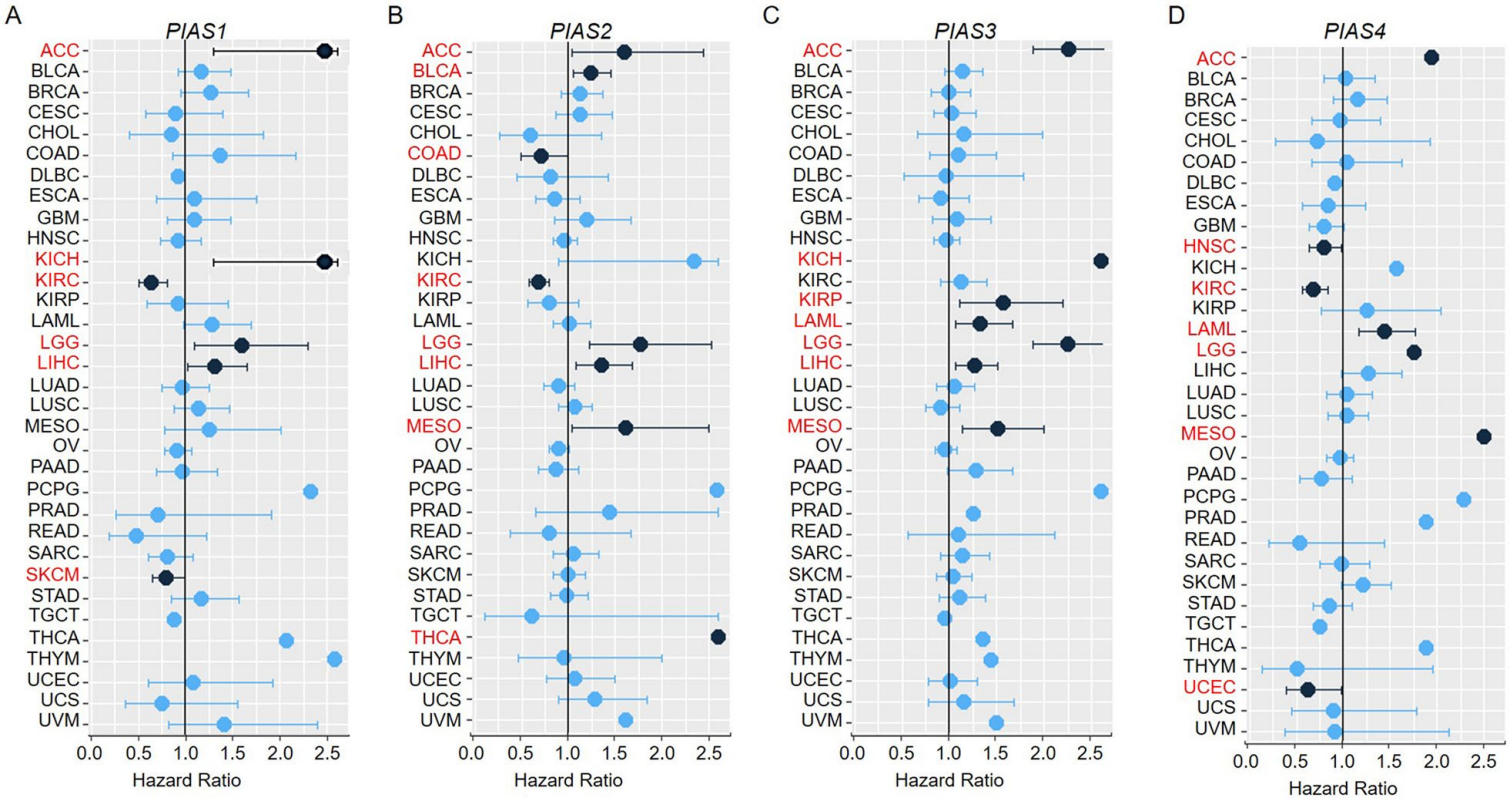
Univariate Cox expression was used to analyze the relationship between PIAS family genes expression and overall survival in thirty-three tumor patients. **A** Correlation of *PIAS1* expression with Cox analysis in different cancer types. **B** Cox regression analysis of *PIAS2* expression in different tumor types. **C** Correlation of *PIAS3* expression with Cox analysis in different cancer types. **D** Cox regression analysis of *PIAS4* expression in different tumor types. Red letters and black dots indicate that the gene is a risk factor in the corresponding tumor

Next, we used a univariate Cox regression model to assess the relationship between PIAS family genes expression and overall survival (OS) in thirty-three cancers from the TGCA dataset. The results showed that *PIAS1* acted as a protective prognostic factor in KIRC and SKCM (HR < 1, p < 0.05), and played a detrimental prognostic factor in ACC, KICH, LGG and LIHC (HR > 1, p < 0.05, Fig. 2A). *PIAS2* was a protective factor for COAD and KIRC (HR < 1, p < 0.05), and played a risk factor for ACC, BLCA, LGG, LIHC, MESO or THCA (HR > 1, p < 0.05, Fig. 2B). During the risk regression analysis, we also found that *PIAS3* was the high-risk gene in ACC, KICH, KIRP, LAML, LGG, LIHC or MESO (HR > 1, p < 0.05, Fig. 2C). *PIAS4* acted as a protective prognostic factor in HNSC, KIRC or UCEC (HR < 1, p < 0.05, Fig. 2D), but was a detrimental prognostic factor in ACC, LAML, LGG, MESO (HR > 1, p < 0.05, Fig. 2D). Additionally, we also explored the correlation of PIAS family genes expression with prognosis in the prognostic scan database. As shown in Table 2, *PIAS1* played a disadvantage prognostic factor in colorectal cancer (DFS). In contrast, *PIAS1* acted as a protective role in breast cancer (DFS, RFS), Head and neck cancer (RFS), and Lung cancer (RFS). *PIAS2* was a protective effect on the prognosis of bladder cancer (OS, DSS), colorectal cancer (DFS, DSS) and brain cancer (OS), but acted as a disadvantage prognostic factor in breast cancer (RFS), lung cancer (OS, RFS) and skin cancer (OS). We also found that *PIAS3* played a deleterious prognostic factor in colorectal cancer (OS, DSS, DFS), while *PIAS4* was a risk factor for breast cancer (DFS, RFS), lung cancer (OS) and brain cancer (OS).

**Table 2:** PIAS family gene expression was related to the prognosis of different cancers in PrognoScan

Gene	Dataset	Cancer type	Endpoint	Number	COX p-value	HR [95% CI^low^—CI^upp^]
*PIAS1*	GSE2034	Breast cancer	DFS	286	0.0042	0.41 [0.22–0.75]
*PIAS1*	GSE2837	Head and neck cancer	RFS	28	0.0059	0.35 [0.16–0.74]
*PIAS1*	GSE31210	Lung cancer	RFS	204	0.0067	0.22 [0.07–0.66]
*PIAS1*	GSE17537	Colorectal cancer	DFS	55	0.0120	5.71 [1.46–22.37]
*PIAS1*	GSE7378	Breast cancer	DFS	54	0.0430	0.11 [0.01–0.93]
*PIAS2*	GSE12276	Breast cancer	RFS	204	0.000044	2.41 [1.58–3.68]
*PIAS2*	GSE12276	Breast cancer	RFS	204	0.0012	1.83 [1.27–2.63]
*PIAS2*	GSE17710	Lung cancer	RFS	56	0.0032	2.11 [1.28–3.46]
*PIAS2*	GSE13507	Bladder cancer	OS	165	0.0052	0.37 [0.18–0.74]
*PIAS2*	GSE17537	Colorectal cancer	DFS	55	0.0067	0.02 [0.00–0.32]
*PIAS2*	GSE17710	Lung cancer	OS	56	0.0086	1.93 [1.18–3.16]
*PIAS2*	GSE17710	Lung cancer	RFS	56	0.0104	1.92 [1.17–3.16]
*PIAS2*	GSE13507	Bladder cancer	DSS	165	0.0106	0.26 [0.09–0.73]
*PIAS2*	GSE12276	Breast cancer	RFS	204	0.0111	1.54 [1.10–2.14]
*PIAS2*	GSE31210	Lung cancer	RFS	204	0.0143	3.10 [1.25–7.67]
*PIAS2*	GSE16581	Brain cancer	OS	67	0.0171	0.03 [0.00–0.55]
*PIAS2*	GSE17710	Lung cancer	OS	56	0.0224	1.74 [1.08–2.79]
*PIAS2*	GSE17537	Colorectal cancer	DSS	49	0.0261	0.01 [0.00–0.61]
*PIAS2*	GSE19234	Skin cancer	OS	38	0.0369	2.67 [1.06–6.73]
*PIAS2*	GSE31210	Lung cancer	OS	204	0.0387	3.91 [1.07–14.24]
*PIAS3*	GSE14333	Colorectal cancer	DFS	226	0.0017	2.40 [1.39–4.16]
*PIAS3*	GSE17536	Colorectal cancer	DSS	177	0.0080	2.19 [1.23–3.91]
*PIAS3*	GSE17536	Colorectal cancer	OS	177	0.0296	1.79 [1.06–3.01]
*PIAS4*	GSE7378	Breast cancer	DFS	54	0.0206	6.39 [1.33–30.71]
*PIAS4*	GSE9195	Breast cancer	RFS	77	0.0317	3.05 [1.10–8.42]
*PIAS4*	GSE13213	Lung cancer	OS	117	0.0354	1.85 [1.04–3.27]
*PIAS4*	GSE9195	Breast cancer	DFS	77	0.0417	3.28 [1.05–10.32]
*PIAS4*	GSE7696	Brain cancer	OS	70	0.0449	3.25 [1.03–10.26]

Annotation: *OS* overall survival, *DFS* Disease Free Survival), *DSS* disease specific survival, *RFS* relapse free survival, *HR* (hazard ratio), *CI* Confidence Interval

**Fig. 3:**
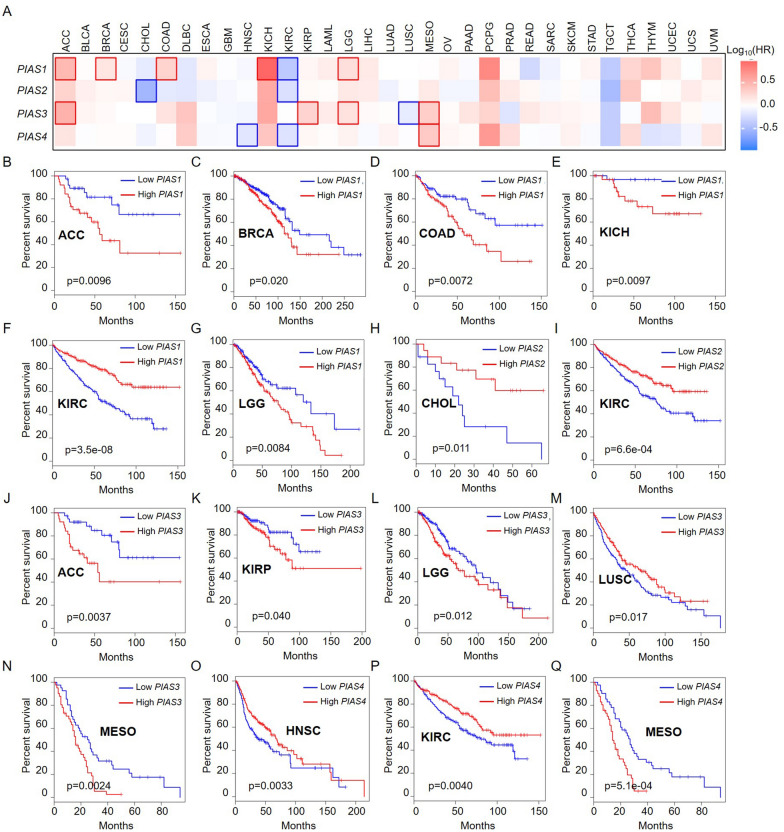
Correlation analysis of PIAS family genes expression and overall survival in patients with different TCGA tumor types. **A** GEPIA2 was used to construct survival profiles of the PIAS family genes expression. Overall survival curves of *PIAS1* in different tumors: **B** ACC; **C** BRCA; **D** COAD; **E** KICH; **F** KIRC; **G **LGG. The overall survival curves of *PIAS2* in different tumors: **H** CHOL; **I** KIRC. Overall survival curves of *PIAS3* in different tumors: **J **ACC; K KIRP; **L** LGG; **M** LUSC; **N** MESO. The overall survival curves of *PIAS4* in different tumors: **O** HNSC; **P** KIRC; **Q** MESO

We further evaluated the prognostic significance of PIAS family genes across pan-cancer. The results suggested that the expression of PIAS family members was associated with the prognosis (OS) of several TCGA types of cancer (Fig. 3A). Kaplan–Meier overall survival curve showed that *PIAS1* played an adverse role in ACC (p = 0.0096, Fig. 3B), BRCA (p = 0.020, Fig. 3C), COAD (p = 0.0072, Fig. 3D), KICH (p = 0.0097, Fig. 3E) and LGG (p = 0.0084, Fig. 3G). Conversely, *PIAS1* was a protective factor on KIRC (p = 3.5e−08, Fig. 3F). *PIAS2* had a protective role in CHOL (p = 0.011, Fig. 3H) and KIRC (p = 6.6e−04, Fig. 3I). *PIAS3* acted as an adverse effect in ACC (p = 0.0037, Fig. 3J), KIRP (p = 0.040, Fig. 3K), LGG (p = 0.012, Fig. 3L) and MESO (p = 0.0024, Fig. 3N). but had a protective role in LUSC (p = 0.017, Fig. 3M). We found that *PIAS4* had an adverse effect on HNSC (p = 0.0033, Fig. 3O) and KIRC (p = 0.0040, Fig. 3P), while acted as a protective factor in MESO (p = 5.1e−04, Fig. 3Q).

**Fig. 4:**
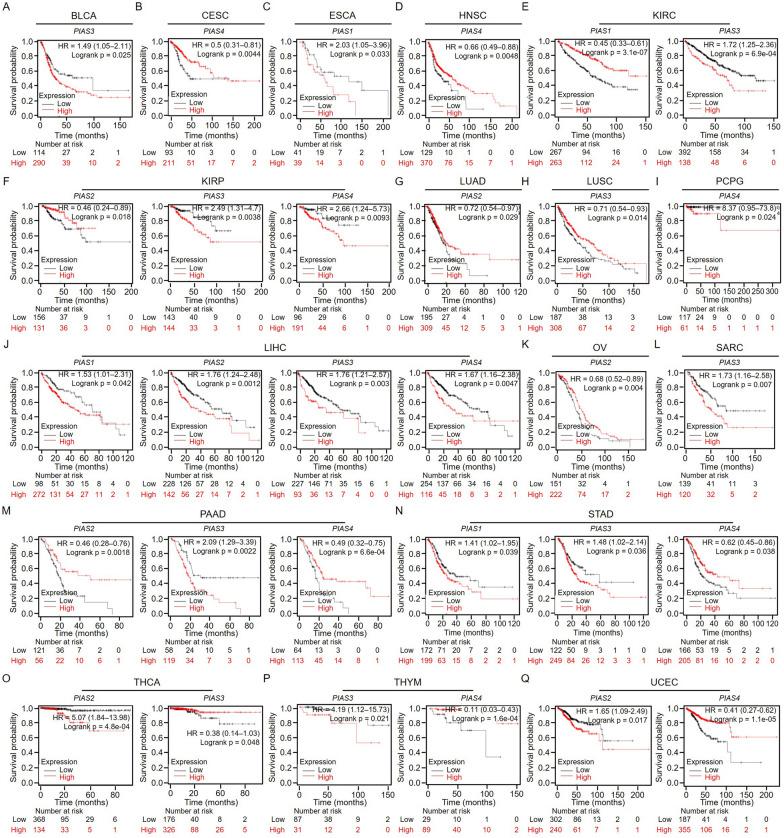
Overall survival curves comparison of high or low expression of PIAS family genes in pan-cancer by using Kaplan–Meier Plotter database. **A** *PIAS3* in BLCA, n = 485; **B** *PIAS4* in CESC, n = 394; **C** *PIAS1* in ESCA, n = 182; **D** *PIAS4* in HNSC, n = 182; **E** *PIAS1, PIAS2* in KIRC, n = 777; **F** *PIAS2*, *PIAS3*, *PIAS4* in KIRP, n = 373; **G** *PIAS2* in LUAD, n = 601; **H** *PIAS3* in LUSC, n = 632; **I** *PIAS4* in PCPG, n = 234; **J** *PIAS1, PIAS2*, *PIAS3*, *PIAS4* in LIHC, n = 704; **K** *PIAS2* in OV, n = 503; **L** *PIAS3* in SARC, n = 353; **M** *PIAS2*, *PIAS3*, *PIAS4* in PAAD, n = 261; **N** *PIAS1*, *PIAS3*, *PIAS4* in STAD, n = 564; **O** *PIAS2*, *PIAS3* in THCA, n = 671; **P** *PIAS3*, *PIAS4* in THYM, n = 485. **Q** *PIAS2*, *PIAS4* in UCEC, n = 713

Furthermore, we also used Kaplan–Meier Plotter online database to explore the correlation between PIAS family gene expression and pan-cancer prognosis (OS). Survival curves showed that *PIAS3* was a poor prognostic gene in BLCA (n = 485, HR > 1, p < 0.05, Fig. 4A). For CESC, *PIAS4* was a low-risk gene (n = 394, HR < 1, p < 0.05, Fig. 4B), and *PIAS1* was a high-risk factor in ESCA (n = 182, HR > 1, p < 0.05, Fig. 4C), as well as *PIAS4* was a protective prognostic factor in HNSC (n = 612, HR < 1, p < 0.05, Fig. 4D). Additionally, we also found that *PIAS1* was a low-risk gene of KIRC (n = 777, HR < 1, p < 0.05), while *PIAS3* was a high-risk gene of KIRC (n = 777, HR > 1, p < 0.05, Fig. 4E). In KIRP, *PIAS2* was a prognostic protective factor (n = 373, HR < 1, p < 0.05), while *PIAS3* (n = 373, HR > 1, p < 0.05) and *PIAS4* (n = 373, HR > 1, p < 0.05) were prognostic risk factors (Fig. 4F). *PIAS2* and *PIAS3* showed protective effects in LUAD (n = 601, HR < 1, p < 0.05, Fig. 4G) and LUSC (n = 632, HR < 1, p < 0.05, Fig. 4H), respectively. Conversely, *PIAS4* showed deleterious effects in PCPG (n = 234, HR > 1, p < 0.05, Fig. 4I). We also found that *PIAS1*, *PIAS2*, *PIAS3*, and *PIAS4* were a prognostic risk effect on LIHC (n = 601, HR < 1, p < 0.05, Fig. 4J). *PIAS2* showed a protective effect in OV (n = 503, HR < 1, p < 0.05, Fig. 4K), and *PIAS3* acted as an adverse effect in SARC (n = 353, HR > 1, p < 0.05, Fig. 4L). During the prognostic analysis, we also found that *PIAS3* showed an adverse role in PAAD (n = 261, HR > 1, p < 0.05), while *PIAS2* and *PIAS4* played a protective effect (n = 261, HR < 1, p < 0.05, Fig. 4L). In STAD, both *PIAS1* and *PIAS3* were a high-risk gene (n = 524, HR > 1, p < 0.05), and *PIAS4* had a prognostic protective effect (n = 524, HR < 1, p < 0.05, Fig. 4M). For THCA, *PIAS2* was a high-risk gene (n = 671, HR > 1, p < 0.05), while *PIAS3* was a low-risk gene (n = 671, HR < 1, p < 0.05, Fig. 4N). In THYM, *PIAS3* acted as an adverse role (n = 181, HR > 1, p < 0.05), and *PIAS4* showed a protective effect (n = 181, HR < 1, p < 0.05, Fig. 4N). Besides, *PIAS2* was a high-risk gene in UCEC (n = 713, HR > 1, p < 0.05), while *PIAS4* was a low-risk gene (n = 713, HR < 1, p < 0.05, Fig. 4O). We also compared the relationship between PIAS family gene expression and pan-cancer prognosis (OS) in different databases. The results showed that their high expression levels were significantly associated with OS improvement in LIHC and KIRP (the bold value), and this association was consistent across different databases (Table 3).

**Table 3:** The association between high expression of PIAS family genes and over survival of pan-cancer in different databases

Gene	role	Kaplan–Meier plotter OS	TCGA OS	TCGA + GTEx OS	PrognoScan OS
***PIAS1***	Protective	KIRC	KIRC, SKCM	KIRC	
	Detrimental	ESCA, **LIHC**, STAD, UCEC	ACC, KICH, LGG, **LIHC**	ACC, BRCA, COAD, KICH, LGG	
***PIAS2***	Protective	**KIRP**, LUAD, OV, PAAD	COAD, KIRC	CHOL, KIRC	Brain cancer,
	Detrimental	**LIHC**, THCA	ACC, BLCA, LGG, **LIHC**, MESO, THCA		Lung cancer, skin cancer
***PIAS3***	Protective	KIRC, LUSC, THCA		LUSC	
	Detrimental	BLCA, **KIRP**, **LIHC**, SARC, PAAD, STAD, THYM	ACC, KICH, **KIRP**, LAML, LGG, **LIHC**, MESO	ACC, **KIRP**, LGG, MESO	Colorectal cancer
***PIAS4***	Protective	CESC, HNSC, PAAD, STAD, THYM, UCEC	HNSC, KIRC, UCEC	HNSC, KIRC	Lung cancer
	Detrimental	**KIRP**, PCPG, **LIHC**	ACC, LAML, LGG, MESO	MESO	Brain cancer

### The genetic alterations of PIAS family genes across pan-cancers

**Fig. 5:**
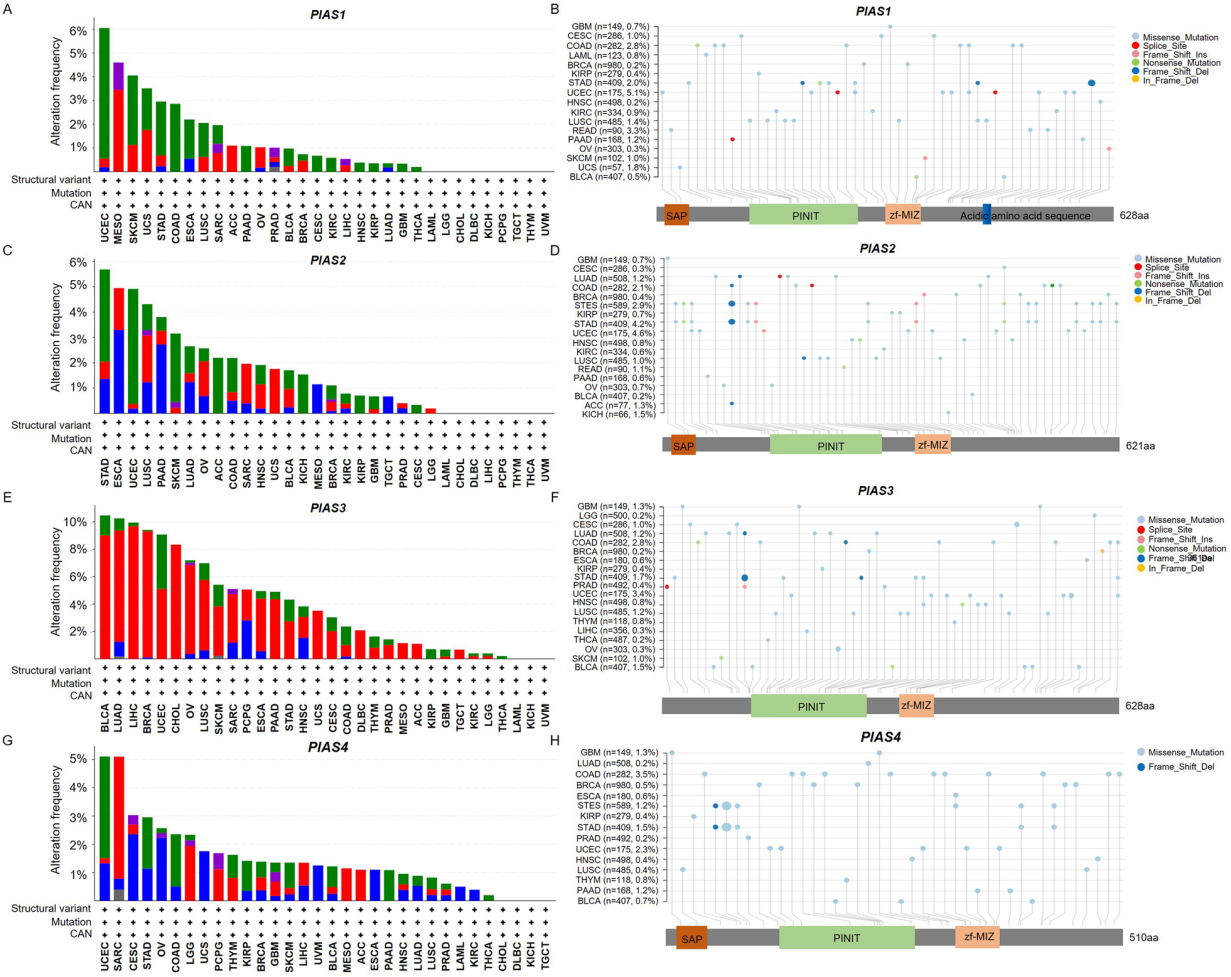
Genetic alterations of PIAS family genes in different types of cancers. **A**–**D** The frequency of PIAS family genes mutations with mutation type across TCGA cancers by cBioPortal. **E**–**H** Mutation sites of PIAS family genes in TCGA samples

**Fig. 6:**
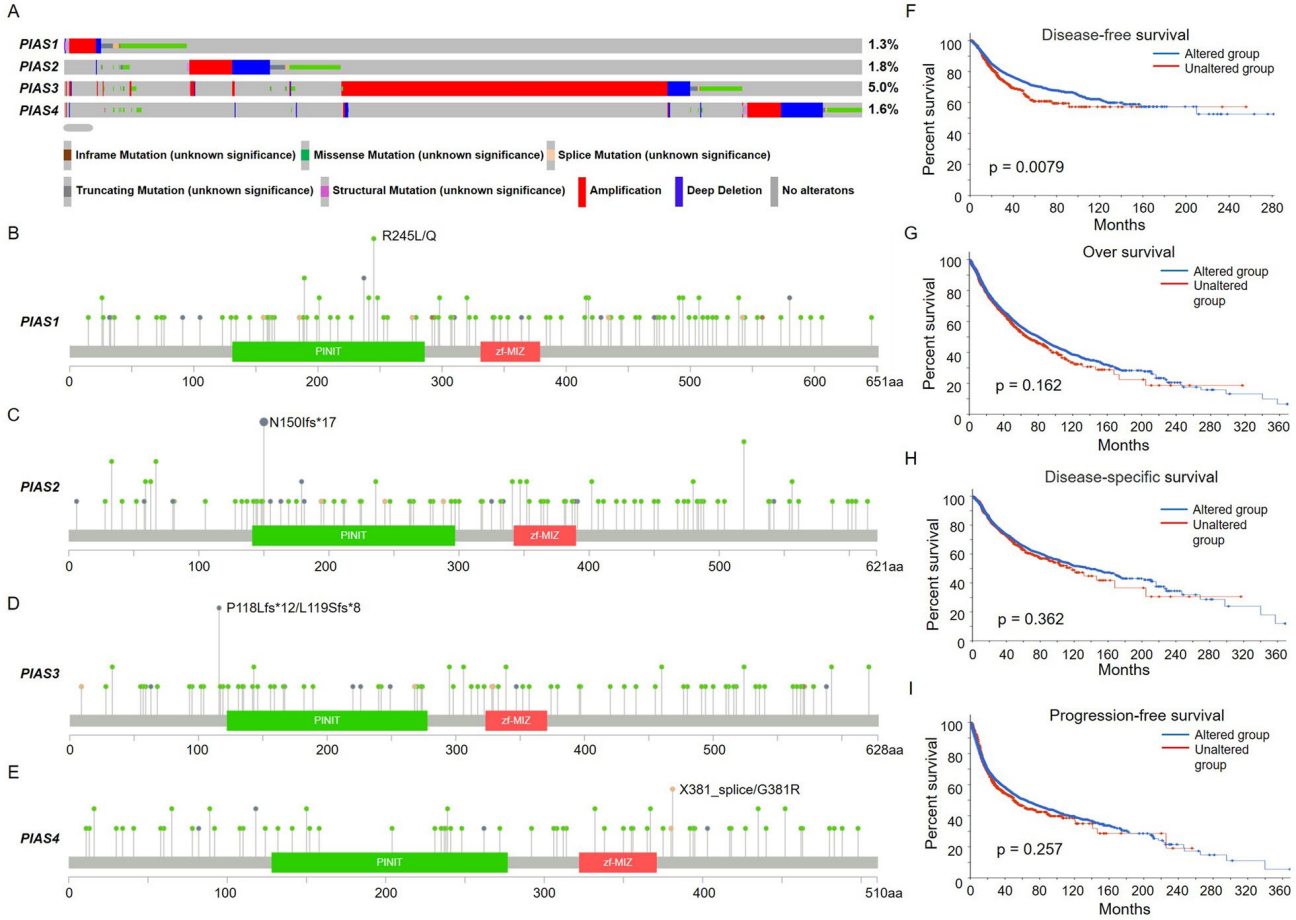
Correlation between genetic alterations of PIAS family genes and prognosis across pan-cancer. **A** Oncoprint of PIAS family genes alterations in cancer cohorts. **B**–**E** Mutation sites of PIAS family genes in pan-cancer data. **F**–**I** The associations of PIAS family genes mutation status with OS, DSS, DFS and PFS in pan-cancer

We attempted to investigate genetic alterations of PIAS family genes in pan-cancer using the cBioportal database. The results showed that they exhibited amplification patterns in most cancer types, and *PIAS1* had the highest amplification pattern in UCES (Fig. 5A, B). *PIAS2* showed the highest amplification pattern in STAD (Fig. 5C, D). In contrast, the amplification pattern of *PIAS3* was the highest in BLCA (Fig. 5E, F), while *PIAS4* had the highest amplification pattern in UCES (Fig. 5G, H). Additionally, the major genetic alterations in PIAS family genes were missense mutations, amplification, and deep deletion (Fig. 6A). During the analysis of genetic alterations, we also found comprehensive data on mutations with important domains of PIAS family genes in the pan-cancer context, which are more frequent. Among them, the R245 site of *PIAS1* had the highest mutation frequency (Fig. 6B), while the N150 site of *PIAS2* showed a higher mutation frequency (Fig. 6C). Conversely, *PIAS3* had the highest mutation frequency at P118 site (Fig. 6D), and the X381 site of *PIAS4* had the highest mutation frequency (Fig. 6E). Subsequently, we also analyzed whether alterations in PIAS family genes affect the prognosis of pan-cancers. The results showed that their mutation was significantly correlated with DFS compared with unaltered (p = 0.0079, Fig. 6F), but not with OS (p = 0.162, Fig. 6G), DSS (p = 0.362, Fig. 6H) and PFS (p = 0.257, Fig. 6I). These findings suggest that genetic alterations in PIAS family genes are associated with poor prognosis of human cancers.

### Association of PIAS family genes expression with TME in pan-cancer

**Fig. 7:**
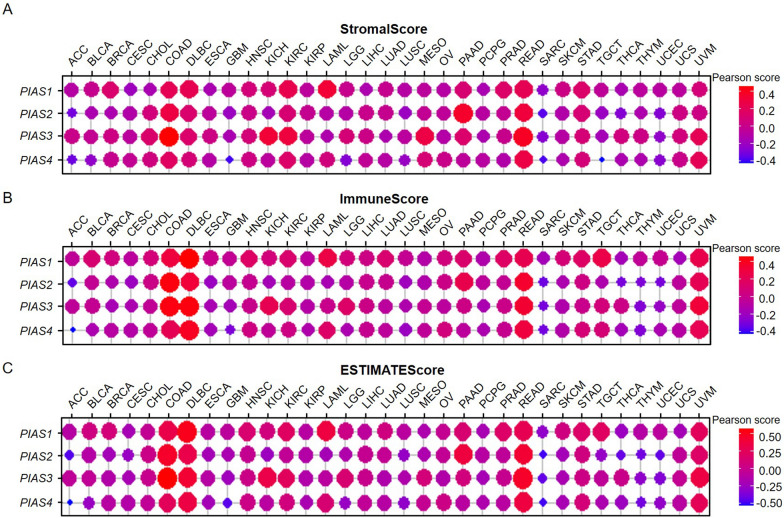
Association of PIAS family genes expression and tumor microenvironment in pan-cancer. **A** The relationship between PIAS gene expression and stromalscore. **B** The relationship between PIAS family gene expression and immunescore. **C** The relationship between PIAS gene expression and ESTIMATEScore. Red dots indicate a positive correlation and blue dots indicate a negative correlation

**Fig. 8:**
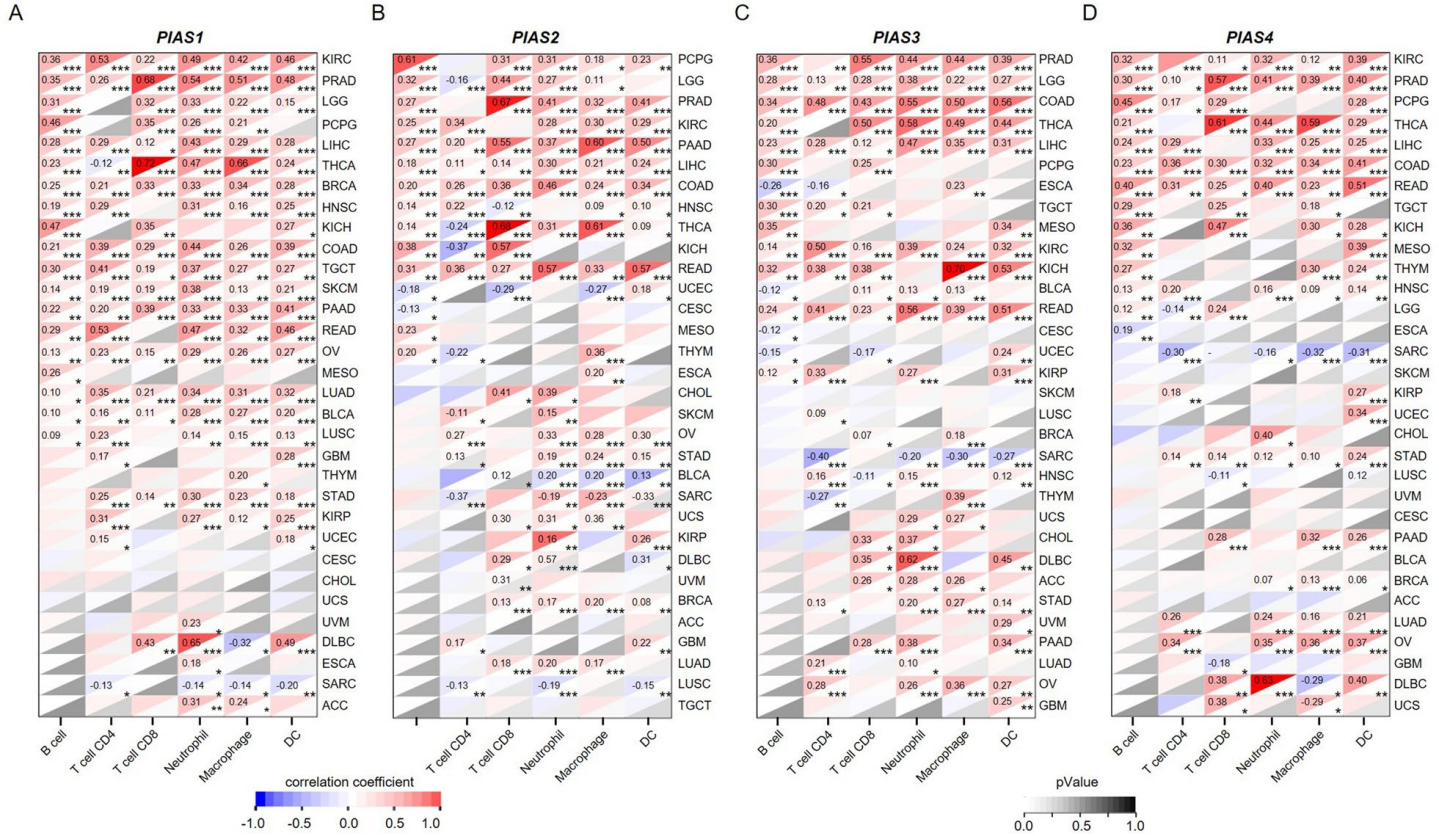
The correlation of PIAS family genes expression with immune cells in pan-caner. **A** The relationship between *PIAS1* expression and different tumor immune cells. **B** The association of *PIAS2* expression with different tumor immune cells. **C** The correlation between *PIAS3* expression and different tumor immune cells. **D** The relationship of *PIAS4* expression with different tumor immune cells. Red background indicates a positive correlation and blue background indicates a negative correlation. *p < 0.05; **p < 0.01; ***p < 0.001

After exploring the relationship between PIAS family genes expression and prognosis, we further evaluated the impact of their expression on the TME in pan-cancer. The results showed that PIAS family genes expression was significantly positively correlated with stromal score (Fig. 7A), immune score (Fig. 7B), and ESTIMATE score (Fig. 7C) in most types of cancer. In addition, we also analyzed the relationship of their expression with immune cells (Fig. 8A–D), including B cell, CD4^+^ T cells CD8^+^ T cell, neutrophils, macrophages, and dendritic cells (DC). These findings suggest that PIAS family genes expression is closely correlation with tumor immune cell infiltration.

**Fig. 9:**
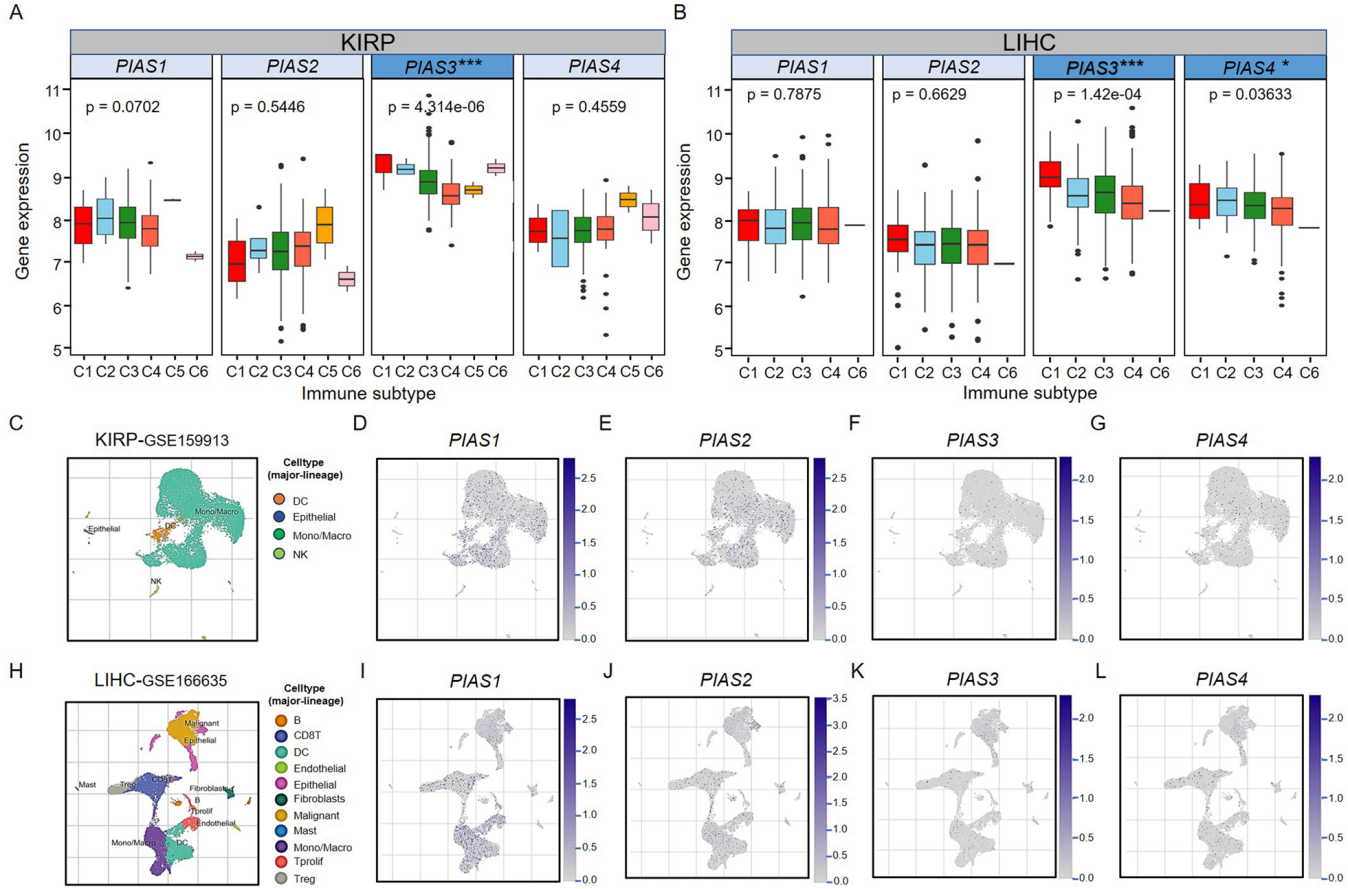
The association of PIAS family genes expression with immune invasive subtypes and immune cells in KIRP and LIHC. **A**, **B** One-way analysis of variance was used to investigate the correlation between PIAS family genes expression and immune invasive subtypes in KIRP and LIHC. **C**–**G** UMAP plots showing cell clusters and PIAS family genes expression levels in different immune cell types in KIRP. **H**–**L** UMAP plots showing cell clusters and PIAS family genes expression levels in different immune cell types in LIHC. *p < 0.05; **p < 0.01; ***p < 0.001

We previously shown that PIAS family genes expression was significantly associated with improved outcomes for LIHC and KIRP in different databases. Subsequently, we focused on the relationship between their expression and immune subtypes in KIRP and LIHC. The samples were divided into six categories according to the type of immune infiltration in the TCGA database, C1 (wound healing), C2 (IFN-g dominance), C3 (inflammation), C4 (lymphocyte depletion), C5 (immunologically quiet), and C6 (TGF-β dominant). By analyzing the relationship between the type of immune infiltration and PIAS family genes expression, we found that *PIAS3* expression was significantly correlated with the immune subtype of KIRP (Fig. 9A). We also found that *PIAS3* and *PIAS4* were significantly associated with immune subtypes in LIHC (Fig. 9B). To explore the potential role of PIAS family genes in TME, we evaluated their expression in different immune cells by single-cell (scRNA) sequencing. We found that they are mainly expressed in monocytes/macrophages in KIRP (Fig. 9C-G) and LIHC (Fig. 9H-L). These findings indicate that PIAS family genes may act as important roles in immunomodulating.

### Association of PIAS family genes expression with stemness score in pan-cancer

**Fig. 10:**
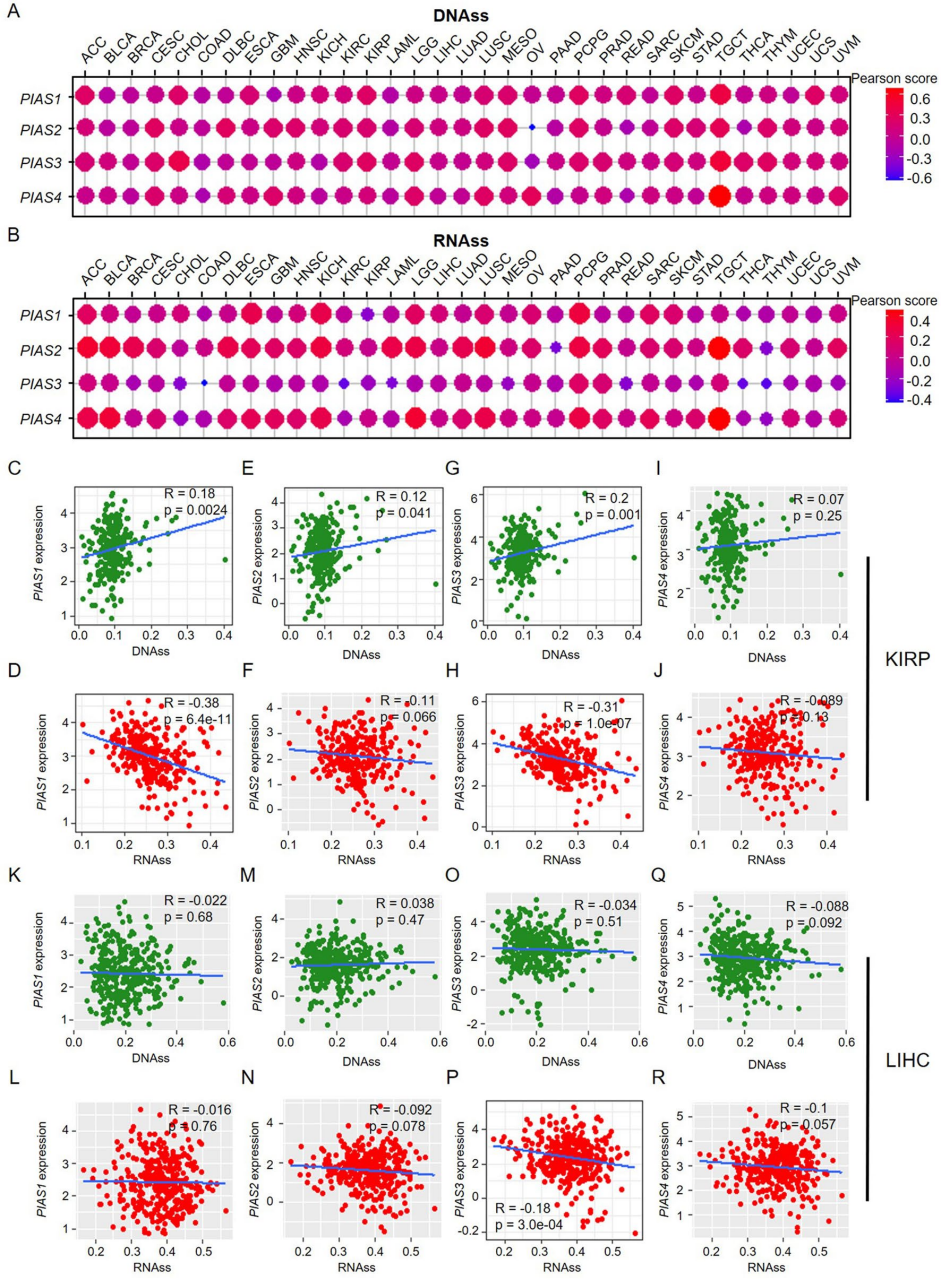
Correlation analysis of PIAS family genes expression with stemness score in different cancers. **A** Relationship between PIAS family genes expression and DNAss. **B** Relationship between PIAS family genes expression and RNAss. **C**–**F** PIAS family genes expression correlated with DNAss in KIRP. **G**–**J** PIAS family genes expression correlated with RNAss in KIRP. **K**–**N** PIAS family genes expression correlated with DNAss in LIHC. **O**–**R** PIAS family genes expression correlated with RNAss in LIHC. Gray background indicates no correlation, and light background indicates that the gene is significantly correlated with the corresponding index. **R** represents correlation value, and p < 0.05 was considered statistically significant

To further explore the effect of PIAS family genes expression on tumor stemness, correlation analysis was performed. In most types of cancer, we found that the expression of PIAS family genes was positively correlated with DNAss (Fig. 10A), and negatively correlated with RNAss (Fig. 10B). In addition, we also found that *PIAS1* expression was significantly positively correlated with DNAss in KIRP (Fig. 10C) and negatively correlated with RNAss (Fig. 10D). *PIAS2* expression was significantly positively correlated with DNAss in KIRP (Fig. 10E), but not with RNAss (Fig. 10F). *PIAS3* expression was significantly positively correlated with DNAss in KIRP (Fig. 10G), and negatively correlated with RNAss (Fig. 10H). *PIAS4* expression was not significantly associated with KIRP DNAss (Fig. 10I) or RNAss (Fig. 10J). During the stemness analysis, we also found no significant correlation of *PIAS1* expression with DNAss (Fig. 10K) and RNAss (Fig. 10L) in LIHC. Similarly, *PIAS2* expression was not correlated with DANss (Fig. 10M) and RNAss (Fig. 10N) in LIHC. *PIAS3* expression was not significantly correlated with DNAss in LIHC (Fig. 10O), but was significantly negatively correlated with RNAss (Fig. 10P). We also observed that *PIAS4* expression showed no significant correlation with DNAss (Fig. 10Q) and RNAss (Fig. 10R) in LIHC. These findings suggest the expression of PIAS family genes is associated with tumor stemness in some human tumors, especially in KIRP and LIHC.

### Effect of PIAS family gene expression on chemotherapy sensitivity

**Fig. 11:**
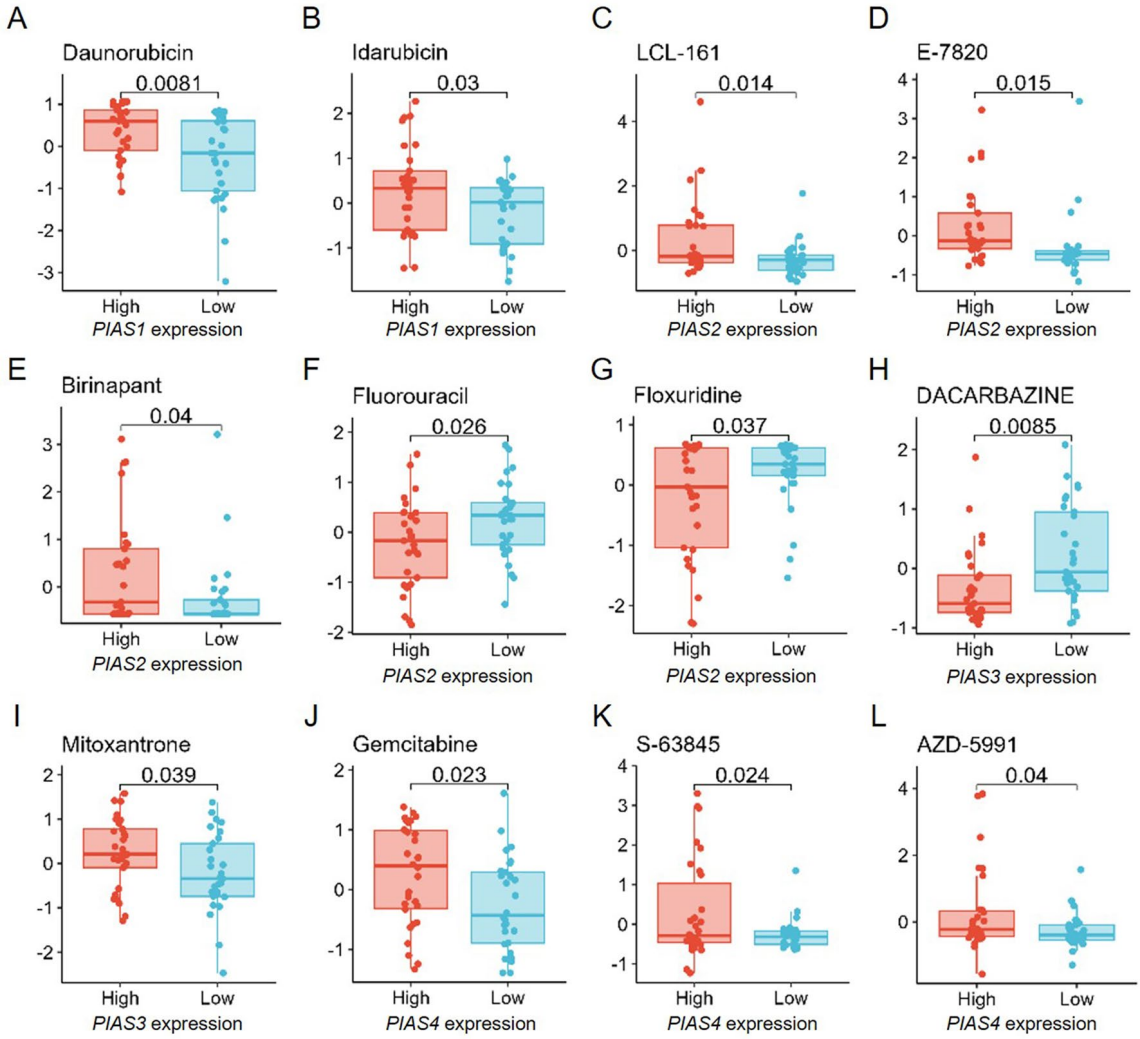
Relationship between PIAS family gene expression and drug sensitivity. *PIAS1* expression was correlated with drug sensitivity of Daunorubicin **A**, Idrubicin **B**; *PIAS2* expression was correlated with drug sensitivity of LCL-161 **C**, E-7820 **D**, Birinapant **E**, Fluorouracil **F**, Floxuridine **G**; *PIAS3* expression was correlated with drug sensitivity of DACARBAZINE **H**, Mitoxantrone **I**; *PIAS4* expression was correlated with sensitivity of Gemcitabine **J**, S-63845 **K**, AZD-5991 **L**. p < 0.05 was considered statistically significant

We have previously identified the predictive role of PIAS family genes expression in pan-cancer. Therefore, it is very appropriate to explore the correlation of their expression with drug sensitivity in human cancer cells. In the sensitivity evaluation of more than 200 chemotherapeutic agents, we analyzed the potential association of PIAS family genes expression with drug sensitivity in 60 human cancer cell lines (NCI-60). The results showed *PIAS1* expression was positively correlated with drug sensitivity of Daunorubicin (Fig. 11A) and Idrubicin (Fig. 11B). *PIAS2* expression was positively correlated with drug sensitivity of LCL-161 (Fig. 11C), E-7820 (Fig. 11D), and Birinapant (Fig. 11E), but negatively correlated with the drug sensitivity of Fluorouracil (Fig. 11F) and Floxuridine (Fig. 11G). *PIAS3* expression was negatively correlated with drug sensitivity of DACARBAZINE (Fig. 11H), and positively correlated with the drug sensitivity of Mitoxantrone (Fig. 11I). Besides, we also found that *PIAS4* expression was positively correlated with sensitivity of Gemcitabine (Fig. 11J), S-63845 (Fig. 11K), and AZD-5991 (Fig. 11L). These results indicate that the expression of PIAS family genes is closely related to the sensitivity of multiple chemotherapy drugs.

### Correlation of PIAS family genes expression with tumor metastasis

**Fig. 12:**
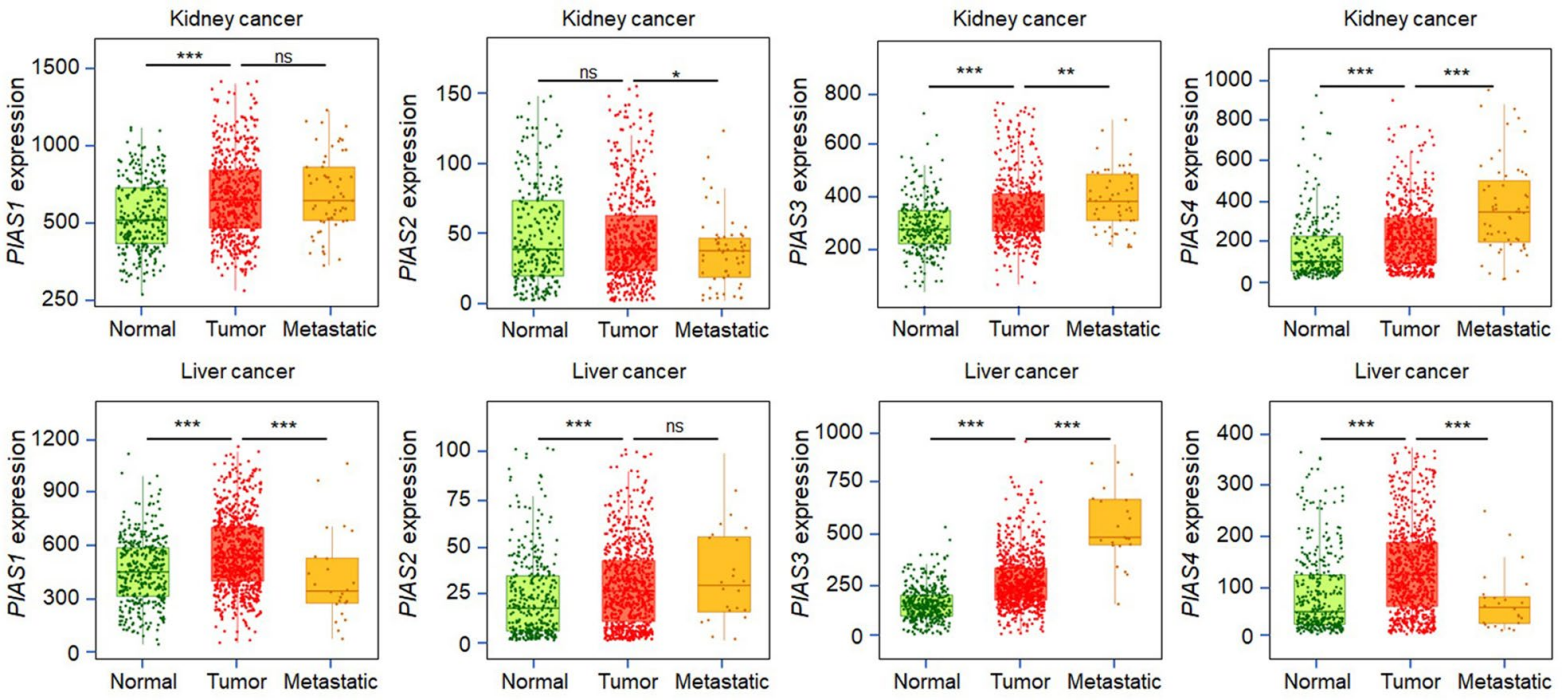
Correlation of PIAS family genes expression and tumor metastasis. **A**–**D** Correlation between PIAS family genes expression and tumor metastasis in kidney cancer. **E**–**H** Differences in the expression levels of PIAS family genes in normal, tumor and metastatic tissues of liver cancer

**Fig. 13:**
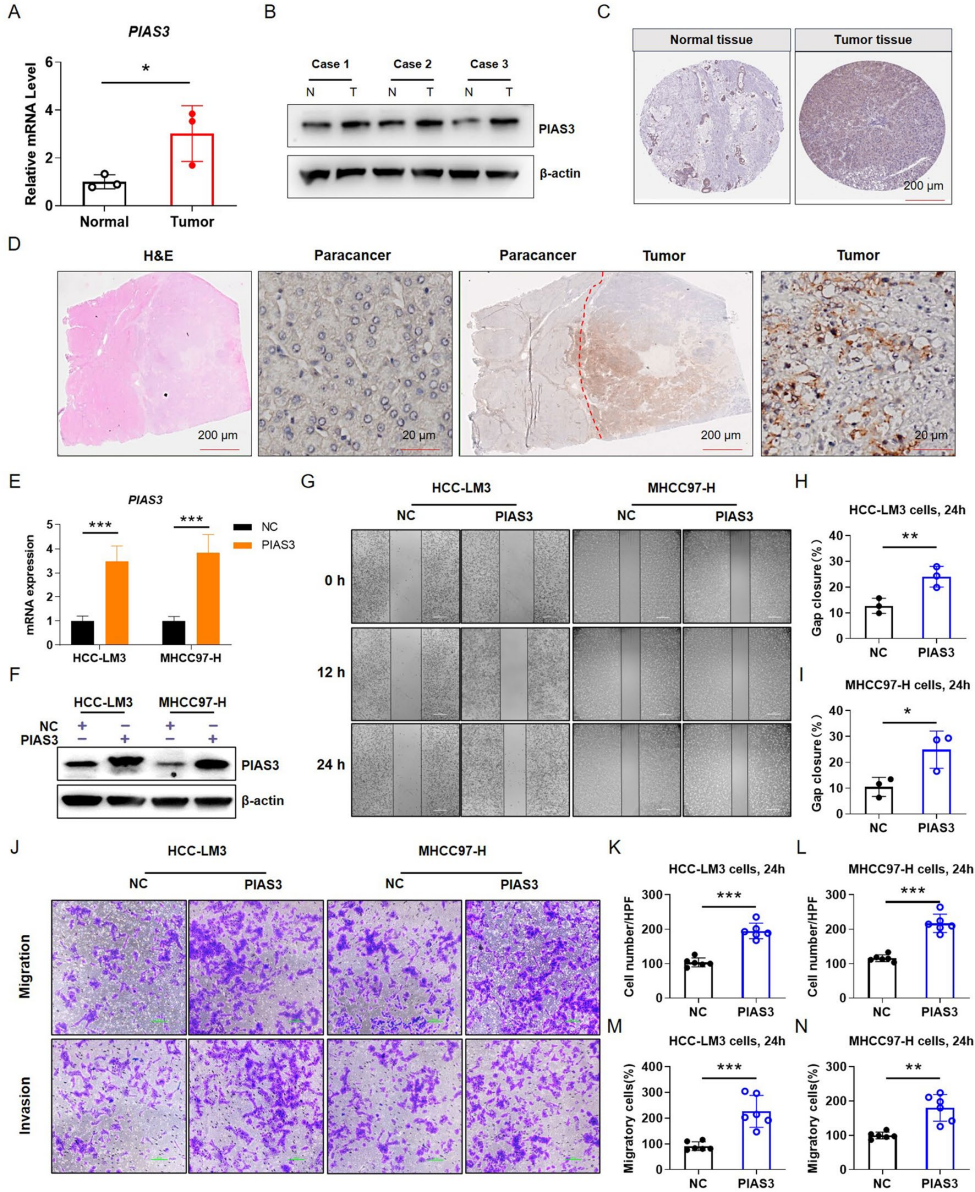
PIAS3 promotes metastasis of HCC-LM3 and MHCC97-H cells. **A** The mRNA expression of *PIAS3* in human liver cancer tissue and normal tissue. **B** The protein level of PIAS3 in human liver cancer tissue and normal tissue. **C** Based on the HPA database, representative immunohistochemical staining of PIAS3 in normal and tumor tissues of LIHC. **D** The expression of PIAS3 protein in human hepatocarcinoma and para-cancerous tissues was analyzed by immunohistochemistry. **E** The mRNA expression of *PIAS3* in HCC-LM3 and MHCC97-H cells. **F** PIAS3 protein expression in HCC-LM3 and MHCC97-H cells. **G** The wound healing assay suggested that *PIAS3* overexpression promoted cell migration of HCC-LM3 and MHCC97-H cells. **H**, **I** Quantitative analysis of wound healing percentage in HCC-LM3 and MHCC97-H cells. **J** The effects of PIAS3 on cell migration and invasion were examined by transwell assays in HCC-LM3 and MHCC97-H cells. (K-N) Quantitative analysis of cell migration and invasion in HCC-LM3 cells and MHCC97-H cells. *p < 0.05;**p < 0.01;***p < 0.001

We previously shown that PIAS family genes expression played important roles in immunomodulating, especially in KIRP and LIHC. Thus, it is significance to further investigate their roles in metastasis of these tumors. We found that the expression of *PIAS3* was significantly positively correlated with metastasis of KIRP (Fig. 12up) and LIHC (Fig. 12down). By analyzing the expression of PIAS family members in human tumor tissues, we found that PIAS3 mRNA (Fig. 13A) and protein (Fig. 13B) expression were more intense in tumor tissues compared to adjacent normal tissues. We also found that the PIAS3 protein is more strongly expressed in tumor tissues by analyzing the actual protein expression in human tumor tissues (Fig. 13C). Subsequently, the similar results in the clinical collection of case tissues by immunohistochemical staining (Fig. 13D). To further investigate the effect of *PIAS3* on invasion of LIHC, transfection experiments were performed. We first overexpressed *PIAS3* plasmids in HCC-LM3 and MHCC97-H cells (Fig. 13E, F). Compared with the control group, overexpression of *PIAS3* significantly accelerated the wound healing rate of HCC-LM3 cells (Fig. 13G, H) and MHCC97-H cells (Fig. 13G, I). Subsequently, transwell assay was used to detect the invasion ability of the cells (Fig. 13J). The results showed that *PIAS3* overexpression enhanced the migration and invasion of HCC-LM3 (Fig. 13K, L) and MHCC97-H cells (Fig. 13M, N). These results suggest that overexpression of *PIAS3* promotes the invasion and migration of liver cancer.

## Discussion

Emerging research focuses on pan-cancer analysis, which was able to reveal the common features of different types of human cancers and facilitate the exploration of new tumor therapeutic targets [23]. In the present study, we performed a systematic pan-cancer analysis to characterize the expression of PIAS family genes in 33 types of human cancers. Furthermore, the correlation of their expression level with prognosis, immune system, TME, tumor stemness, and chemotherapy resistance was evaluated. We explored the possible roles of mutations in the PIAS family genes in pan-cancer, and also revealed their immunological functions in different tumors. Our findings highlight the multiple biological roles of PIAS family genes in a variety of human cancers, providing a new perspective for further investigate on the occurrence and development of tumors.

With the in-depth research of PIAS protein family, it has been found that they have a variety of biological functions, including gene transcription, protein post-translational modification, and signaling pathway activation, etc. [24]. Previous studies have shown that *PIAS1* is increased in human prostate cancer and promotes tumor cell proliferation by inhibiting cell cycle inhibitors [25]. In addition, high *PIAS3* expression promoted the invasion and migration of gastric cancer and was strongly negatively correlated with the survival of patients [26]. These findings highlight the biological roles of PIAS family genes in tumor progression. In this study, we analyzed the expression of PIAS family genes (*PIAS1*, *PIAS2*, *PIAS3*, and *PIAS4*) in 33 different types of cancer. Our results showed that *PIAS1* was significantly downregulated in ten cancer types and significantly upregulated in seven cancer types. *PIAS2* expression was significantly decreased in six cancer types and significantly elevated in six cancer types. *PIAS3* expression was significantly decreased in two cancer types and significantly up-regulated in fifteen cancer types. We also found that *PIAS4* was also significantly down-regulated in two cancer types and up-regulated in fifteen cancer types. These findings are beneficial to determine whether PIAS family genes are a possible oncogenic target gene, and provide a theoretical basis for further investigate of their biological function in tumors.

Compared with surgical treatment, adjuvant chemotherapy improves the survival rate of cancer patients [27]. However, many patients do not benefit from adjuvant chemotherapy because the therapeutic response to chemotherapy drugs is influenced by the interaction between TME cells and their chemokine networks [28, 29]. Traditional tumor staging systems are not good at predicting response to chemotherapy [30], whereas biomarkers have shown the ability to play a useful role in predicting treatment response. Using NCI-60 cell line datasets, we investigated the relationship between PIAS family genes expression and drug sensitivity. Our results showed *PIAS1* expression was positively correlated with drug sensitivity of Daunorubicin and Idrubicin. *PIAS2* expression was positively correlated with drug sensitivity of LCL-161, E-7820, and Birinapant, while negatively correlated with the drug sensitivity of Fluorouracil and Floxuridine. *PIAS3* expression was negatively correlated with drug sensitivity of DACARBAZINE, and positively correlated with the drug sensitivity of Mitoxantrone. *PIAS4* expression was positively correlated with sensitivity of Gemcitabine, S-63845, and AZD-5991. These findings illustrate the potential biological roles of PIAS family genes in the susceptibility or resistance of tumor cells to drug treatment, which will make an important contribution to future investigate on the influence of PIAS family genes for cancer immunotherapy.

Tumor heterogeneity, immune status, and the interrelationship between tumor and stromal cells in the tumor microenvironment may influence therapeutic effectiveness [31]. A disturbed immune microenvironment is significantly associated with tumor progression [32–34]. Furthermore, it has been reported that dysregulation of extracellular matrix (ECM) homeostasis and cell–cell adhesion in TME are key drivers of cancer development [35–37]. The presence of tumor immune cells in TME has been clearly identified as an important prognostic indicator of patient survival and a potential target for tumor therapy [38]. This study found that the expression of PIAS family genes was closely related to immune cell infiltration, especially in KIRP and LIHC. Moreover, inflammatory mediators secreted by immune cells not only promote EMT, but also transform normal epithelial cells into cancer cells by increasing cellular DNA damage and mutation [39]. Previous studies have shown that EMT regulates the invasion and metastasis of tumor cells [40], and promotes the occurrence of multi-drug resistance [41]. These findings and the current results provide sufficient evidence that PIAS family genes may act as important roles in TME through immunomodulation.

The phenotype and genetic characteristics of gene expression are closely related to tumor progression [42]. The specific signaling pathways often acquire activation mutations in many different types of cancer [43]. In some cases, cancer depends on these characteristics and promotes its proliferation, migration, invasion, and metastasis. Pan-cancer analysis of different cancer types provides comprehensive insights into tumor biology and cancer molecular phenotypes, which helps to identify genomic changes that may be a role in carcinogenic phenotypes [44]. In this study, we found that PIAS family genes exhibit amplification patterns in most cancer types. By analyzing the comprehensive data of mutations in important domains of PIAS family genes in the pan-cancer context, we revealed the sites with the highest mutation frequency. We also found that *PIAS3* expression was positively correlated with LIHC metastasis, and demonstrate that *PIAS3* promoted the migration and invasion of HCC-LM3 and MHCC97-H cells. These findings suggest that genetic alterations in PIAS family genes are associated with cancer metastasis in humans, particularly in LIHC.

Based on genomic technology, we revealed the potential biological role of PIAS genes in human pan-cancer from multiple perspectives, such as expression patterns, genetic alterations, immune cell infiltration, tumor stemness and drug sensitivity. Despite the current study may improve the overall understanding of the roles of PIAS family genes in pan-cancer, there are certain limitations. First of all, this research is mainly based on bioinformatics and lacks in-depth molecular mechanism investigation at the cellular or animal level. In addition, PIAS family genes expression is associated with recruitment of tumor-associated immune cell infiltration and poor prognosis, but it is not possible to determine whether PIAS family genes affect clinical survival through immune signaling pathways. In the future, the specific mechanisms by which PIAS family gene expression affects immune cell infiltration should be further identified in tumors to help provide accurate and personalized cancer treatments.

## Conclusions

In summary, our study reveals that the expression of PIAS family genes is associated with poor prognosis of pan-cancer. In addition to modulating the tumor microenvironment and immune cell infiltration, we also found the relationship of their expression with tumor metastasis, particularly in KIRP and LIHC. Furthermore, PIAS family genes expression is associated with sensitivity or resistance to drug therapy in cancer cells. These findings help to determine whether the PIAS family gene is a possible oncogenic target gene, which will make an important contribution to future cancer treatment research targeting PIAS family genes. These findings provide a novel insight into the investigation of PIAS family genes as pan-cancer specific biomarkers, which will make an important contribution to the development of PIAS family genes targeted therapy research.

## Data Availability

All relevant data are available from the authors upon request and the corresponding author will be responsible for replying to the request.
